# Supplementary figures and images for: Classical complement pathway inhibition reduces brain damage in a hypoxic ischemic encephalopathy animal model
Source: PLoS One. 2021 Sep 30;16(9):e0257960. doi: 10.1371/journal.pone.0257960 (PMC8483388; doi:10.1371/journal.pone.0257960)

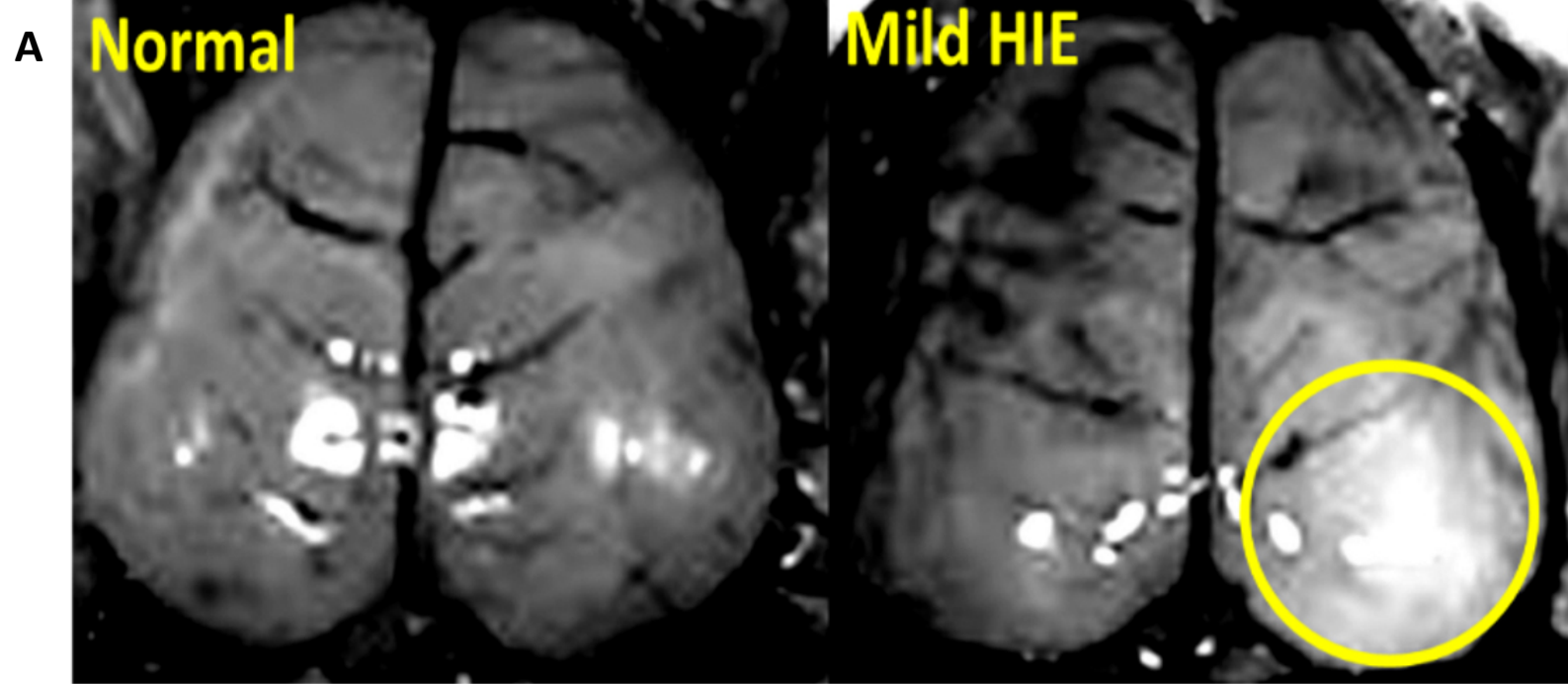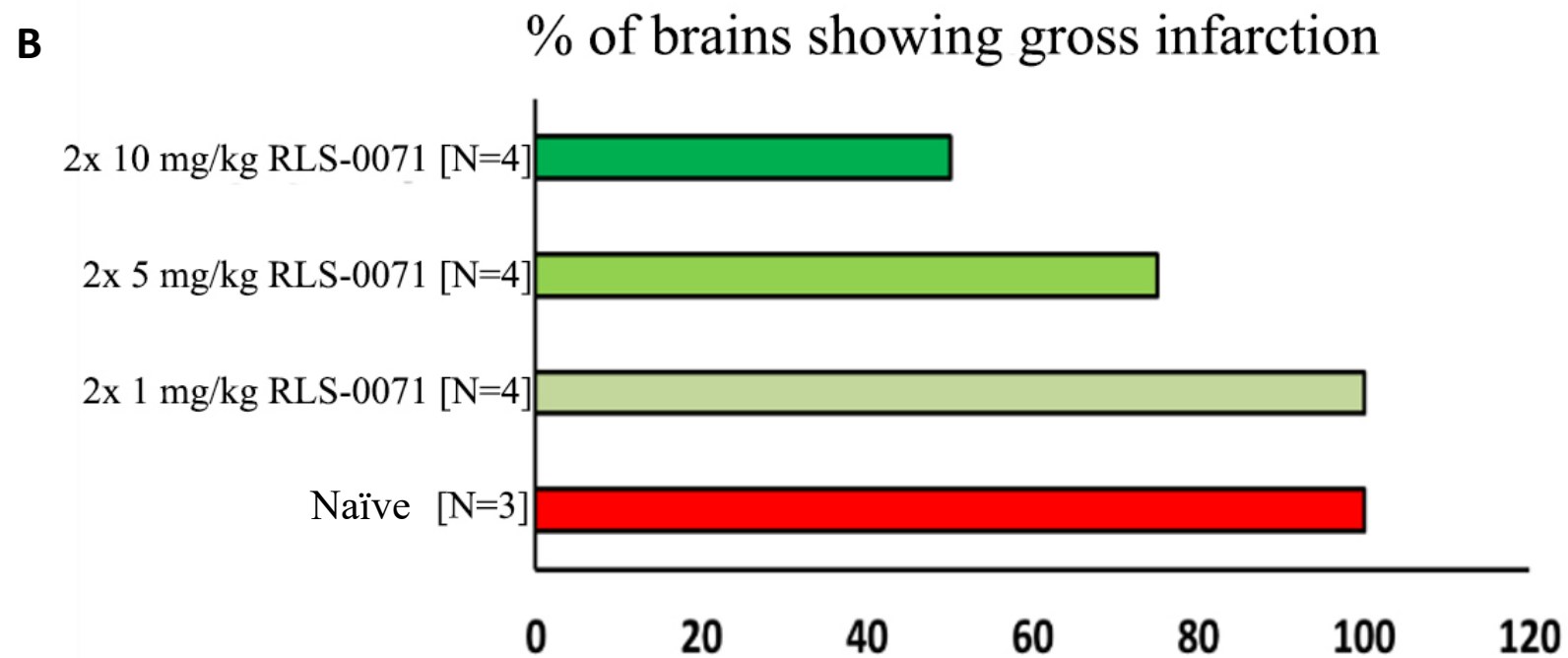

Supplement: S1 Fig — Rat pups were treated with different doses of RLS-0071 (also known as PIC1) at one hour post hypoxia with a repeat dose 4 hours later or untreated (normothermia. NT). RLS-0071 doses: 10 mg/kg × 2, 5 mg/kg × 2, 1 mg/kg × 2 were piloted. Animals were euthanized 48 hours after hypoxia, brains extracted, photographed and evaluated for any evidence of gross brain infarction. Panel A: The image on the left shows a normal appearing brain after treatment with PIC1 (10 mg/kg × 2). The image on the right shows the brain from an animal that did not receive RLS-0071 kept at normothermia (NT). The area of infarction is circled in yellow. Panel B: The graph shows the percent of animals showing any gross evidence of brain infarction for each group. (PDF) [file pone.0257960.s001.pdf]

## Brain C1q Levels

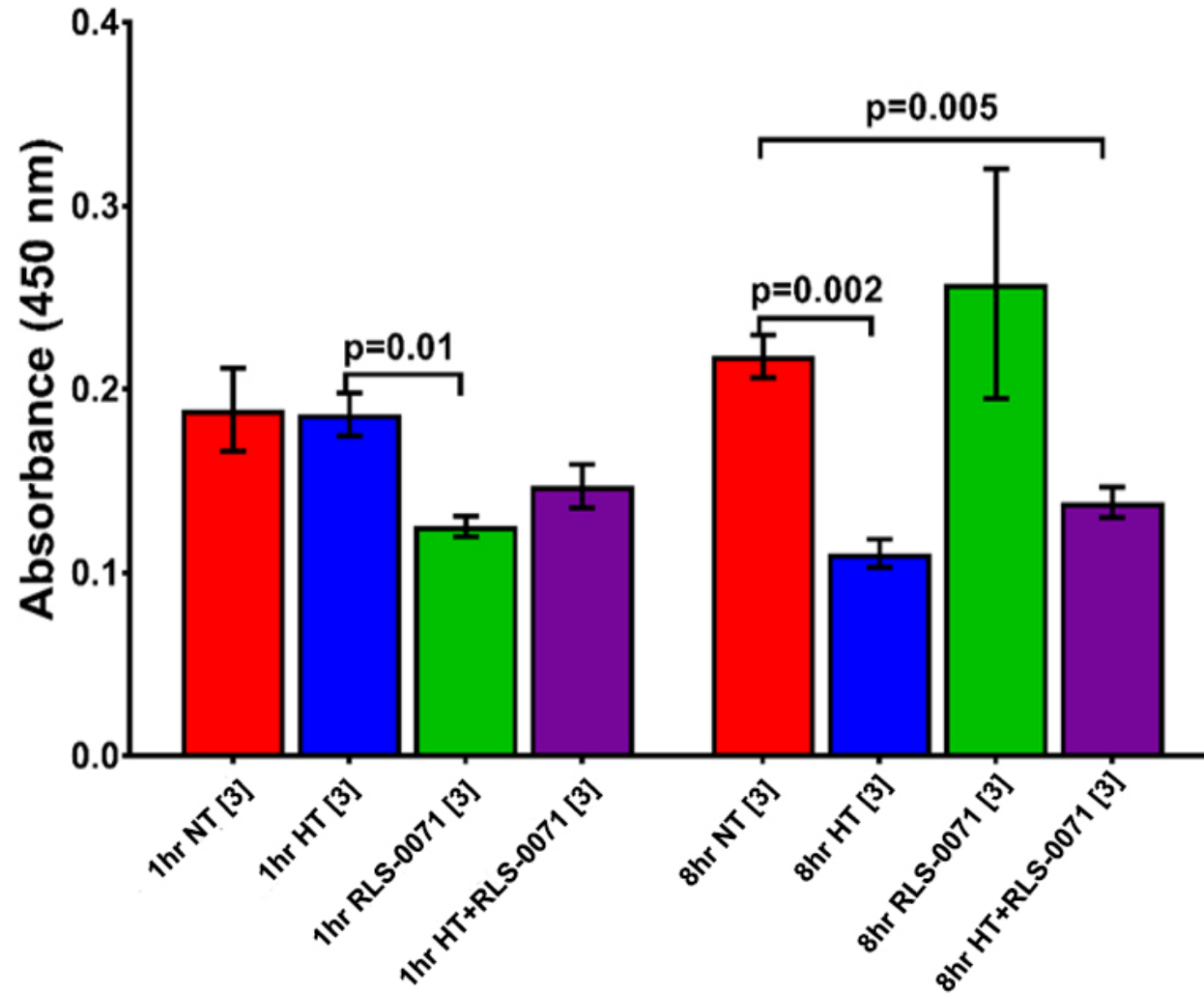

Supplement: S2 Fig — C1q measured by ELISA on brain homogenates were prepared after euthanasia at 1 hour or 8 hours after the second RLS-0071 dosing. Data shown are means ± SEM. Groups: normothermia (NT), hypothermia (HT), RLS-0071 10 mg/kg ×2 doses (RLS-0071), hypothermia and RLS-0071 (HT+RLS-0071). Number of animals are shown in []. (PDF) [file pone.0257960.s002.pdf]

# MRI Data – Representative images of T2 map

NT at 24hr

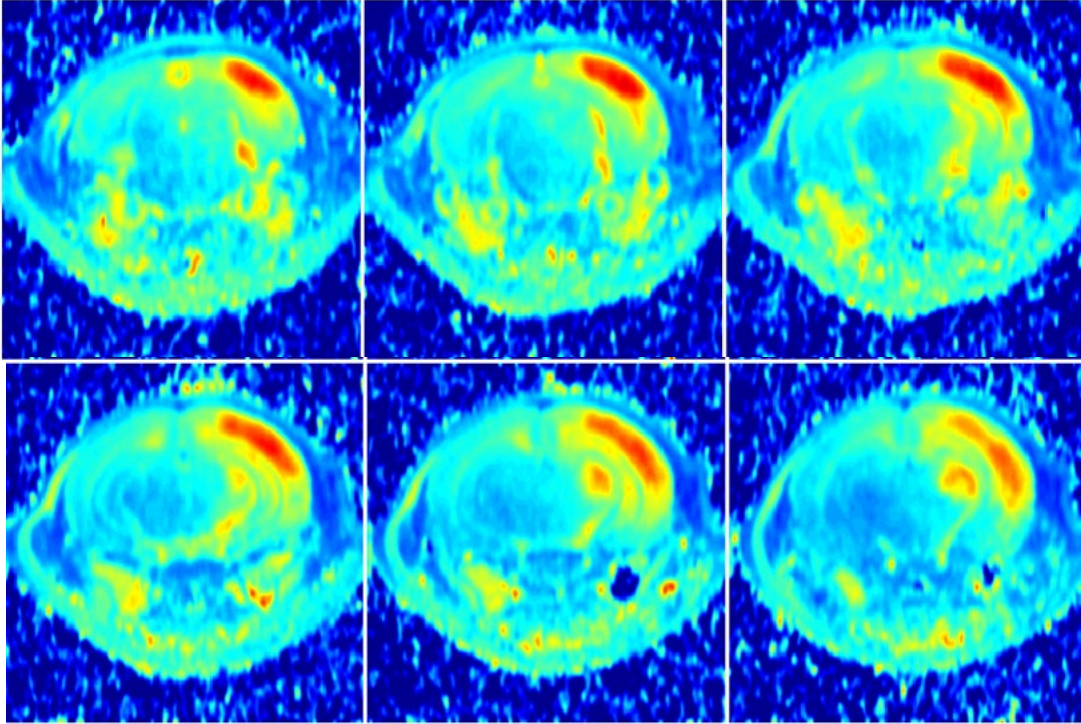

HT+ RLS-0071 at 24hr

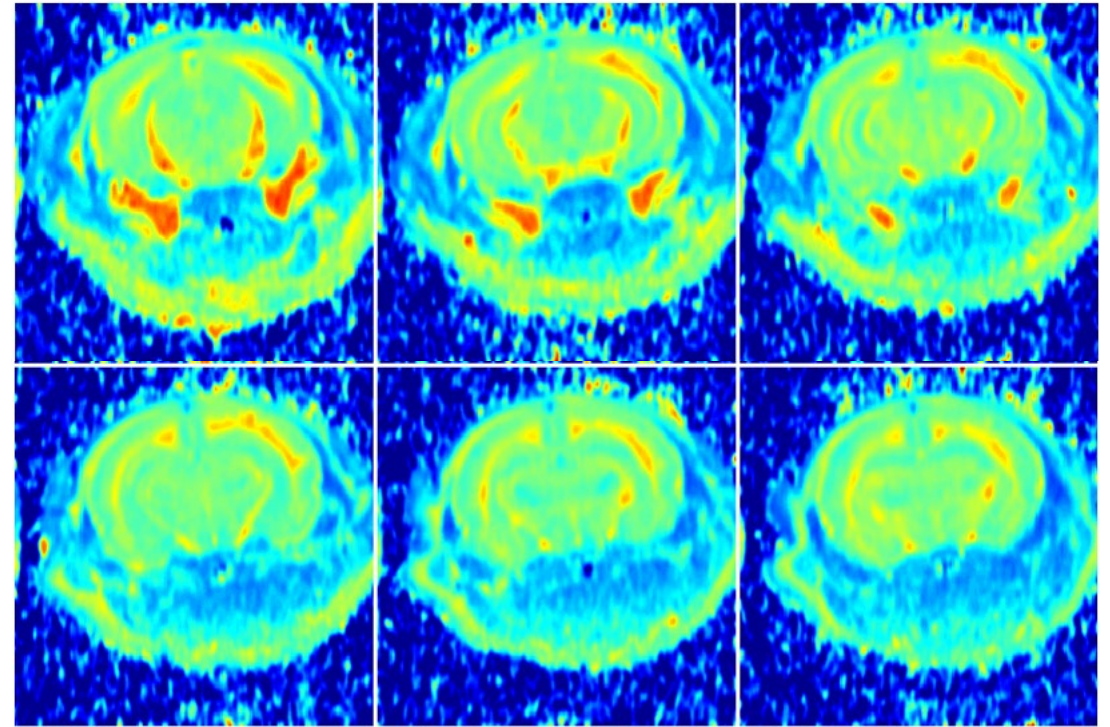

Supplement: S3 Fig — Representative figure of the effects of normothermia and hypothermia +RLS-0071 treatment on lesion T2 values of neonatal Wistar rat pup subjected to brain hypoxia/ischemia (H/I) measured at 24 hours confirm the developed lesion in NT animals. Groups: normothermia (NT) and RLS-0071 with hypothermia (HT+RLS-0071). (PDF) [file pone.0257960.s003.pdf]

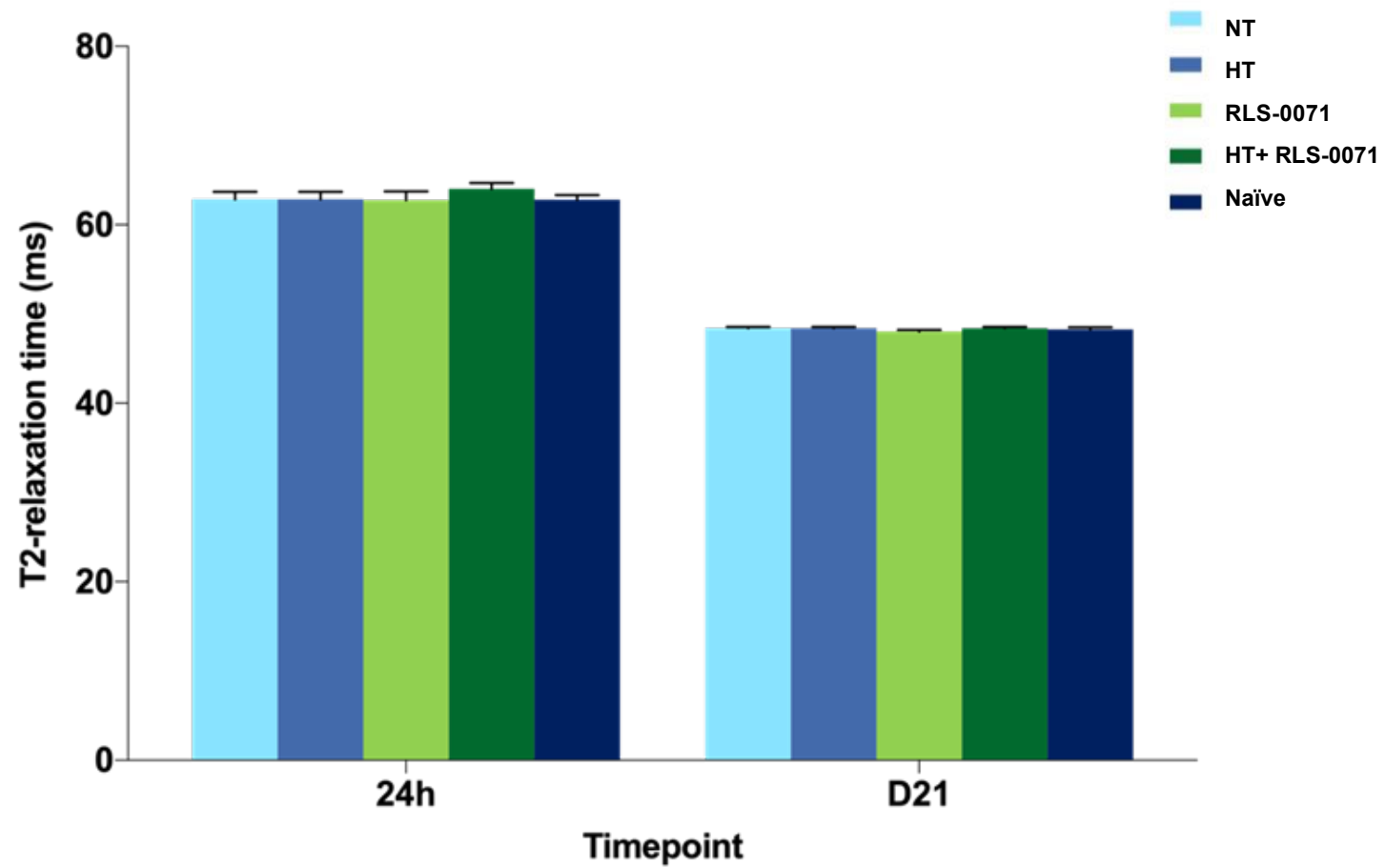

Supplement: S4 Fig — The effects of normothermia, hypothermia and RLS-0071 treatment (with and without hypothermia) on control T2 values of neonatal Wistar rat pups (pooled gender) subjected to brain hypoxia/ischemia (H/I). Data are presented as mean ± SEM. Group 1: NT, n = 9; Group 2: HT, n = 10; Group 3: RLS-0071, n = 9; Group 4: HT+ RLS-0071, n = 9; Naïve, n = 5; n = number of animals. (PDF) [file pone.0257960.s004.pdf]
